# Clinical phenotypes and heath-related quality of life of COPD patients in a rural setting in Malaysia – a cross-sectional study

**DOI:** 10.1186/s12890-020-01295-4

**Published:** 2020-09-29

**Authors:** Chee-Shee Chai, Sumastika Bt Mos, Diana-Leh-Ching Ng, Greta-Miranda-Kim-Choo Goh, Anselm-Ting Su, Muhammad Amin B. Ibrahim, Aisya Natasya Bt Musa, Seng-Beng Tan, Yong-Kek Pang, Chong-Kin Liam

**Affiliations:** 1 Department of Medicine, Faculty of Medicine and Health Science, University Malaysia Sarawak, Kota Samarahan, Sarawak Malaysia; 2 Department of Nursing, Faculty of Medicine and Health Science, University Malaysia Sarawak, Kota Samarahan, Sarawak Malaysia; 3 Department of Community Medicine and Public Health, Faculty of Medicine and Health Science, University Malaysia Sarawak, Kota Samarahan, Sarawak Malaysia; 4 Department of Medicine, Faculty of Medicine, University Technology MARA, Sungai Buloh, Selangor Malaysia; 5 Department of Medicine, Faculty of Medicine, University of Malaya, Kuala Lumpur, Malaysia

**Keywords:** Chronic obstructive pulmonary disease, Clinical phenotypes, Health-related quality of life, Exacerbators, Asthma overlap

## Abstract

**Background:**

The Spanish chronic obstructive pulmonary disease (COPD) guideline phenotypes patients according to the exacerbation frequency and COPD subtypes. In this study, we compared the patients’ health-related quality of life (HRQoL) according to their COPD phenotypes.

**Methods:**

This was a cross-sectional study of COPD patients who attended the outpatient clinic of the Serian Divisional Hospital and Bau District Hospital from 23th January 2018 to 22th January 2019. The HRQoL was assessed using modified Medical Research Council (mMRC), COPD Assessment Test (CAT), and St George’s Respiratory Questionnaire for COPD (SGRQ-c).

**Results:**

Of 185 patients, 108 (58.4%) were non-exacerbators (NON-AE), 51 (27.6%) were frequent exacerbators (AE), and the remaining 26 (14.1%) had asthma-COPD overlap (ACO). Of AE patients, 42 (82.4%) had chronic bronchitis and only 9 (17.6%) had emphysema. Of the 185 COPD patients, 65.9% had exposure to biomass fuel and 69.1% were ex- or current smokers.

The scores of mMRC, CAT, and SGRQ-c were significantly different between COPD phenotypes (*p* <  0.001). There were significantly more patients with mMRC 2–4 among AE (68.6%) (*p* <  0.001), compared to those with ACO (38.5%) and NON-AE (16.7%). AE patients had significantly higher total CAT (*p* = 0.003; *p* <  0.001) and SGRQ-c (both *p* <  0.001) scores than those with ACO and NON-AE. Patients with ACO had significantly higher total CAT and SGRQ-c (both *p* <  0.001) scores than those with NON-AE.

AE patients had significantly higher score in each item of CAT and component of SGRQ-c compared to those with NON-AE (all *p* <  0.001), and ACO [(*p* = 0.003–0.016; *p* = < 0.001–0.005) except CAT 1, 2 and 7. ACO patients had significantly higher score in each item of CAT and component of SGRQ-c (*p* = < 0.001–0.040; *p* <  0.001) except CAT 2 and activity components of SGRQ-c.

**Conclusions:**

The HRQoL of COPD patients was significantly different across different COPD phenotypes. HRQoL was worst in AE, followed by ACO and NON-AE. This study supports phenotyping COPD patients based on their exacerbation frequency and COPD subtypes. The treatment of COPD should be personalised according to these two factors.

## Background

Chronic obstructive pulmonary disease (COPD) is a common, preventable, and treatable airway disease characterized by persistent respiratory symptoms and airflow limitation, due to inflammatory response of the airway and lung tissue to noxious particles or gases [1]. Worldwide, COPD is currently the fourth leading cause of death and is expected to rank number three by 2030 [2]. It also ranks second in the disease burden measured by the disability-adjusted life-years, [3] causing substantial socioeconomic burden in many countries [1].

COPD phenotype is defined as a single or combination of disease attributes that describe the differences between individuals with COPD according to their clinically meaningful outcomes, such as exacerbation, symptoms, rate of disease progression, response to therapy, and mortality risk [4]. The idea of conceptualizing different COPD phenotypes came from Snider in 1989, when “chronic bronchitis”, “emphysema” and “asthmatic” were presented in three overlapping circles in a non-proportional Venn diagram [5]. In 2012, the Spanish Society of Pulmonology and Thoracic Surgery proposed to phenotype COPD based on the exacerbation frequency and existing COPD subtypes [6].

Health-related quality of life (HRQoL) is defined as an individual’s happiness or satisfaction with an aspect of his/her life which is affected by physical, mental, emotional and social health [7]. Impaired HRQoL is common in COPD patients due to the troublesome respiratory symptoms, limited physical activity, psychological distress, sleep disturbance and concomitant co-morbidities [8]. While there have been many studies to determine the impact of COPD on the patients’ HRQoL, studies that specifically compare HRQoL across different COPD phenotypes are limited, particularly in Asian countries and in the rural setting.

In this study, we aimed to compare the HRQoL of patients with COPD attending the hospitals in rural area of Malaysia based on their clinical phenotype. We hypothesize that COPD patients with frequent exacerbation and chronic bronchitis have the worst HRQoL.

## Methods

### Study design and patients

We conducted a cross-sectional study on patients with COPD attending the outpatient clinics of the Serian Divisional Hospital and Bau District Hospital from 23th January 2018 to 22th January 2019. Both hospitals are primary care centres that serve the rural population of southern Sarawak, a state in Malaysia located in northern Borneo Island. All patients were aged 35 years and above, with the ratio of post-bronchodilator forced expiratory volume in one second (PB-FEV_1_) to post-bronchodilator forced vital capacity in six seconds (PB-FVC_6_) <  0.7. Patients with clinical or radiological diagnosis of other chronic lung diseases (such as bronchiectasis and interstitial lung disease), active tuberculosis and lung tumours were excluded. The estimated minimum sample size for the study was 140 based on the prevalence of 10.1% in previous study at 5% of Type-1 error and 5% of precision [9, 10]. The primary objective of this study was to compare the modified Medical Research Council (mMRC) score, total COPD Assessment Tool (CAT) score and total St George’s Respiratory Questionnaire COPD (SGRQ-c) score of patients with different COPD phenotypes. The score of each item of CAT and each component of SGRQ-c of different COPD phenotypes were compared as a secondary objective. Written informed consent was obtained from every patient. Ethics approval was obtained from the Medical Research and Ethics Committee of the National Medical Research Registry of Malaysia (NMRR-17-2549-38,621) and the respective hospitals. The study was conducted in accordance to the Declaration of Helsinki.

### Procedure

We consecutively identified eligible patients from the outpatient clinics of both hospitals. Patient demographic and clinical data were acquired from face-to-face interview and the case notes.

Never-smokers were individuals who had smoked < 100 cigarettes in their lifetime [11]. Ex-smokers and current-smokers were defined as those who smoked ≥100 cigarettes in their lifetime, the former having quitted smoking for at least a year at the time of interview [11]. Biomass exposure was defined as exposure to biomass smoke from the burning of wood or charcoal for ≥100 h per year [12]. PB-FEV_1_ was expressed in percent of predicted value based on the patients’ age, gender, height, and ethnicity (PB-FEV_1_% predicted) [13]. A severe exacerbation was defined as an exacerbation that required hospital admission, while a moderate exacerbation was defined as an exacerbation that required outpatient treatment with corticosteroids and/or antibiotic [14]. The total number of exacerbations in this study only included severe and moderate exacerbations because these types of exacerbations and not mild ones are associated with an increased risk of future exacerbations [1]. The phenotypes of COPD were defined according to the Spanish COPD guideline (GesPOC) 2017 [15]. A non-exacerbator phenotype (NON-AE) was defined as having no severe exacerbation and ≤ one episode of moderate exacerbation in the past 1 year. Exacerbator phenotype (AE) was defined as having any severe exacerbation or ≥ two episodes of moderate exacerbations in the past 1 year. AE was further divided into chronic bronchitis (AE-CB) and emphysema (AE NON-CB). The former was defined by the presence of cough and sputum for ≥ 3 months in a year for two consecutive years [16]; while the latter was defined by the presence of air-trapping on examination or investigations [17]. Asthma-COPD overlap phenotype (ACO) included patients who had previously been diagnosed as bronchial asthma (BA); or had PB-FEV_1_ ≥ 15% and ≥ 400 ml improvement over pre-bronchodilator FEV_1_; or blood eosinophil ≥300 cells/mm [3, 18].

The patients’ HRQoL was assessed using mMRC, CAT and SGRQ-c questionnaires. Patients answered these questionnaires independently in original English version, or validated Malay/Chinese version. They could obtain explanation from the investigators if there was any problem with understanding the questionnaires. mMRC only measured the severity of dyspnea: no dyspnea except on strenuous activity – 0; dyspnea when walking uphill – 1; walked slower than people of the same age because of dyspnea – 2; dyspnea after walking 100 m on level ground and needed to stop for breath – 3; and dyspnea when dressing or too dyspnoeic to leave home – 4 [19]. mMRC 0–1 was defined as low symptom, while 2–4 was defined as high symptom [1]. Eight items, each with score ranging 0–5 are measured in the CAT questionnaire. These included cough – CAT 1; sputum – CAT 2; chest tightness – CAT 3; dyspnea – CAT 4; activity limitation – CAT 5; confidence to leave home – CAT 6; sleep – CAT 7; and energy - CAT 8 [20]. The total CAT score in normal individuals is ≤6 [21]. The SGRQ-c questionnaire consists of three components. The symptoms component consists of questions 1–7; activity component consists of questions 9 and 12; and impact component consists of questions 8, 10, 11, 13 and 14 [22]. The total score of SGRQ-c, as well as the score of each component range 0–100%. The SGRQ-c score for normal individuals is ≤6%; symptoms component ≤12%; activity component ≤9%; and impact components ≤2% [23]. For all questionnaires, higher scores denote poorer HRQoL.

### Statistical analysis

Categorical variables are presented as percentages. The difference between clinical phenotypes was compared using the chi-squared test, with post-hoc analysis taking adjusted standardized residual of > 2 as significant. Continuous variables are presented as the mean ± standard deviation (SD), or median with inter-quantile range. Differences between clinical phenotypes were compared using one-way ANOVA test or Kruskall-Wallis H test. The post-hoc analysis was performed using Tukey test or Dunn’s procedure with a Bonferroni adjustment, respectively. The significant *p*-value in this study was < 0.05. Statistical analyses were performed using the software package, Statistical Package for the Social Sciences (SPSS for Windows version 23.0, SPSS Inc., Chicago, IL, USA).

## Results

### Demographic and clinical characteristics

**Fig. 1:**
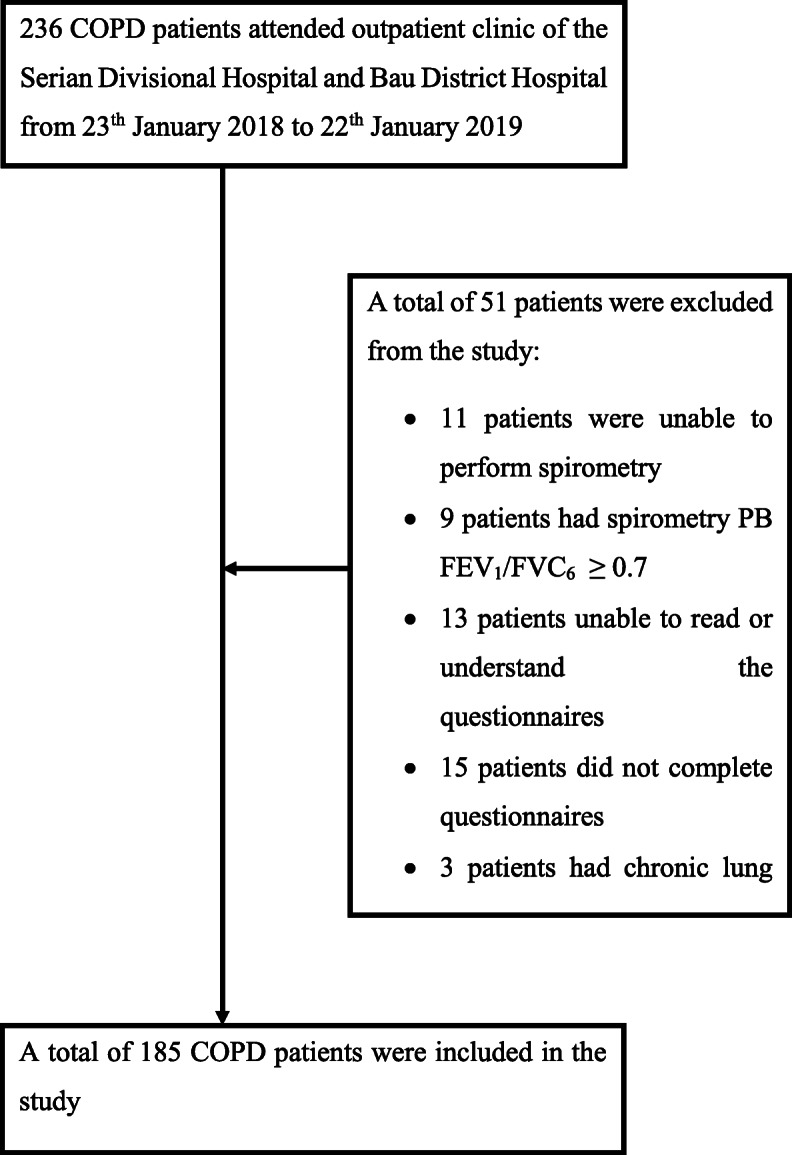
Algorithm of patients’ recruitment in the study

We included 185 patients in this study (Fig. 1). Their demographic and clinical characteristics are described in Table 1. Patients were mostly males, natives of the state of Sarawak, current or ex-smokers and had biomass fuel exposure.

**Table 1:** Demographic and clinical characteristics of 185 patients according to COPD phenotypes

Characteristic	No. of patients, n 185	COPD phenotype, n (%)			
		NON-AE 108 (58.4)	ACO 26 (14.1)	AE 51 (27.6)	*p*-value
**Age, years (mean ± SD, 95% CI)**	62.5 ±11.9;	60.5 ± 11.6;	60.5 ± 14.0;	67.9 ± 10.0;	**0.001**
	60.8 − 64.3	58.3–62.7	54.9–66.2	65.1–70.7	
**Gender, n (%)**					
Male	142 (76.8)	83 (76.9)	15 (57.7)	44 (86.3)	**0.019**
Female	43 (23.2)	25 (23.1)	11 (42.3)	7 (13.7)	
**Ethnicity, n (%)**					
Malay	31 (16.8)	12 (11.1)	6 (23.1)	13 (25.5)	0.161
Chinese	20 (10.8)	14 (13.0)	2 (7.7)	4 (7.8)	
Native of the state of Sarawak	134 (72.4)	82 (75.9)	18 (69.2)	34 (66.7)	
**Smoking status, n (%)**					
Never smoker	57 (30.9)	35 (32.4)	11 (42.3)	11 (21.6)	0.151
Ex- or current smoker	128 (69.1)	73 (67.6)	15 (57.7)	40 (78.4)	
**Biomass fuel exposure, n (%)**					0.249
No	63 (34.1)	34 (31.5)	7 (26.9)	22 (43.1)	
Yes	122 (65.9)	74 (68.5)	19 (73.1)	29 (56.9)	
**Risk for COPD, n (%)**					0.430
Cigarette smoking	63 (34.1)	34 (31.5)	7 (26.9)	22 (43.1)	
Biomass fuel exposure	49 (26.5)	31 (28.7)	10 (38.5)	8 (15.7)	
Both	73 (39.4)	43 (39.8)	9 (34.6)	21 (41.2)	
**Smoking intensity, pack-years (mean ± SD, 95% CI)**	17.1 ±16.9;	14.3 ± 14.2;	18.1 ± 19.6;	22.5 ± 19.5;	**0.016**
	14.7 − 19.6	11.6–17.0	10.2–26.0	17.0–28.0	
**PB- FEV**_**1**_**, % (mean ± SD, 95% CI)**	42.8 ±19.5;	43.6 ± 20.1;	44.4 ± 17.9;	40.4 ± 19.3.	0.566
	40.0–45.7	39.8–47.4	37.2–51.7	34.9–45.8	
**Exacerbations, episode (mean ± SD, 95% CI)**					
Total	1.4 ±2.7;	0.2 ± 0.4;	0.2 ± 0.4;	4.7 ± 3.4;	**< 0.001**
	1.0–1.8	0.1–0.3	0.1–0.4	3.8–5.7	
Moderate	1.1 ±2.2;	0.2 ± 0.4;	0.2 ± 0.4;	3.6 ± 2.9;	**< 0.001**
	0.8 − 1.4	0.1–0.3	0.1–0.4	2.8–4.4	
Severe	0.3 ±0.8;	0	0	1.1 ± 1.2;	**< 0.001**
	0.2 − 0.4	–	–	0.8–1.5	

*Abbreviation*: *COPD* chronic obstructive pulmonary disease, *NON-AE* non-exacerbators, *ACO* asthma-COPD overlap, *AE* frequent exacerbators, *PB-FEV*_*1*_ post bronchodilator forced expiratory volume in 1 s, *SD* standard deviation; 95% CI, 95% confidence interval

*p*-values with bold are significant

One hundred and eight (58.4%) patients belonged to the NON-AE phenotype, 51 (27.6%) patients were AE phenotype, and the remaining 26 (14.1%) patients had ACO. Of AE patients, 42 (82.4%) had chronic bronchitis and only 9 (17.6%) had lung emphysema. AE patients were significantly older than those with ACO (67.9 ± 10.0 versus 60.5 ± 14.0 years, *p* = 0.024) or NON-AE (67.9 ± 10.0 versus 60.5 ± 11.6 years, *p* = 0.001). The smoking intensity in terms of pack-years of AE patients was significantly higher than that of NON-AE patients (22.5 ± 19.5 versus 14.3 ± 14.2 pack years, *p* = 0.012), but not significantly different from that of ACO patients. There were significantly more female patients with ACO (42.3%) compared to AE (13.7%) or NON-AE (23.1%) (*p* = 0.019). The total exacerbation episodes of ACO patients were significantly lower than that of AE patients (0.2 ± 0.4 versus 4.7 ± 3.4, *p* <  0.001). Otherwise, there was no significant difference in ethnicity, smoking status, biomass exposure, and PB-FEV_1_ between the COPD phenotypes.

The mean scores of mMRC, CAT, and SGRQ-c were significantly different across different COPD phenotypes (all *p* <  0.001) (Table 2). A significantly higher percentage of AE patients had mMRC 2–4 (68.6%), compared to ACO patients (38.5%) and NON-AE patients (16.7%). Patients with AE had significantly higher total CAT and SGRQ-c scores than those with ACO (17.3 ± 9.5 versus 11.7 ± 8.6, *p* = 0.003; 53.5 ± 22.7% versus 34.4 ± 19.5%, *p* <  0.001) and NON-AE (17.3 ± 9.5 versus 5.5 ± 4.7, *p* <  0.001; 53.5 ± 22.7% versus 16.4 ± 14.8%, *p* <  0.001). Patients with ACO also had significantly higher total CAT and SGRQ-c scores than those with NON-AE (11.7 ± 8.6 versus 5.5 ± 4.7, *p* <  0.001; 34.4 ± 19.5% versus 16.4 ± 14.8%, *p* <  0.001).

**Table 2:** mMRC, CAT and SGRQ-c scores of COPD patients according to their COPD phenotypes

Quality of Life Measurement	Clinical Phenotype, n (%)			
	NON-AE 108 (58.4)	ACO 26 (14.1)	AE 51 (27.6)	*p-*value
**mMRC, n, (%)**				
0–1	90 (83.3)	16 (61.5)	16 (31.4)	**< 0.001**
2–4	18 (16.7)	10 (38.5)	35 (68.6)	
**CAT, score (mean ± SD, 95% CI)**				
Total	5.5 ± 4.7;	11.7 ± 8.6;	17.3 ± 9.5;	**< 0.001**
	4.6–6.4	8.2–15.2	14.6–19.9	
Cough	1.9 ± 1.3;	2.6 ± 1.1;	3.2 ± 1.5;	**< 0.001**
	1.7–2.2	2.2–3.1	2.8–3.6	
Mucus	1.3 ± 1.2;	1.9 ± 1.5;	2.5 ± 1.6;	**< 0.001**
	1.0–1.5	1.3–2.5	2.1–3.0	
Chest tightness	0.4 ± 0.7;	1.4 ± 1.4;	2.2 ± 1.4;	**< 0.001**
	0.3–0.5	0.9–2.0	1.9–2.6	
Walk uphill	0.9 ± 1.1;	1.8 ± 1.3;	2.7 ± 1.5;	**< 0.001**
	0.7–1.1	1.3–2.3	2.3–3.1	
Home activity	0.3 ± 0.7;	1.2 ± 1.5;	2.0 ± 1.6;	**< 0.001**
	0.2–0.5	0.6–1.8	1.5–2.4	
Leaving home	0.2 ± 0.6;	0.9 ± 1.1;	1.7 ± 1.5;	**< 0.001**
	0.1–0.3	0.4–1.3	1.2–2.1	
Sleep	0.2 ± 0.6;	0.9 ± 1.2;	1.2 ± 1.2;	**< 0.001**
	0.1–0.3	0.4–1.4	0.9–1.6	
Energy	0.3 ± 0.6;	1.0 ± 1.4;	1.8 ± 1.5;	**< 0.001**
	0.2–0.4	0.5–1.6	1.3–2.2	
**SGRQ-c, % (mean ± SD, 95% CI)**				
Total	16.4 ± 14.8;	34.4 ± 19.5;	53.5 ± 22.7;	**< 0.001**
	13.5–19.2	26.5–42.2	47.1–59.8	
Symptoms	18.3 ± 14.3;	41.9 ± 16.1;	64.6 ±20.2;	**< 0.001**
	15.5–21.0	35.4–48.4	58.9 − 70.3	
Activities	27.1 ± 23.2;	36.3 ± 19.4;	57.8 ± 20.9;	**< 0.001**
	22.6–31.5	28.4–44.1	52.0–63.7	
Impact	9.3 ± 14.3;	30.6 ± 26.1;	47.1 ± 29.9;	**< 0.001**
	6.6–12.1	20.0–41.2	38.7–55.5	

*Abbreviation*: *COPD* chronic obstructive pulmonary disease, *NON-AE* non-exacerbators, *ACO* asthma-COPD overlap, *AE* frequent exacerbators, *mMRC* modified Medical Research Council, *CAT* COPD Assessment Test, *SGRQ-c* St George’s Respiratory Questionnaire for COPD, *SD* standard deviation; 95% CI, 95% confidence interval

*p*-values with bold are significant

**Fig. 2:**
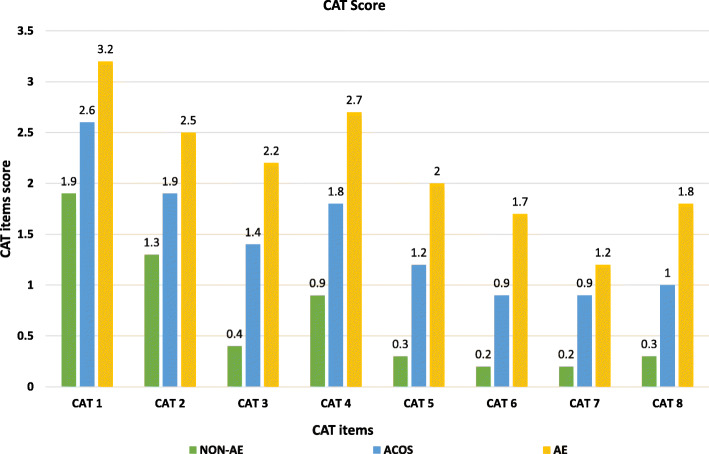
Score of CAT items according to the COPD phenotypes. Abbreviation: CAT, COPD Assessment Test; NON-AE, non-exacerbators; ACO, Asthma-COPD overlap; AE, frequent exacerbators; CAT 1, cough; CAT 2, sputum; CAT 3, chest tightness; CAT 4, dyspnea; CAT 5, activity limitation; CAT 6, confidence to leave home; CAT 7, sleep; CAT 8, energy

**Fig. 3:**
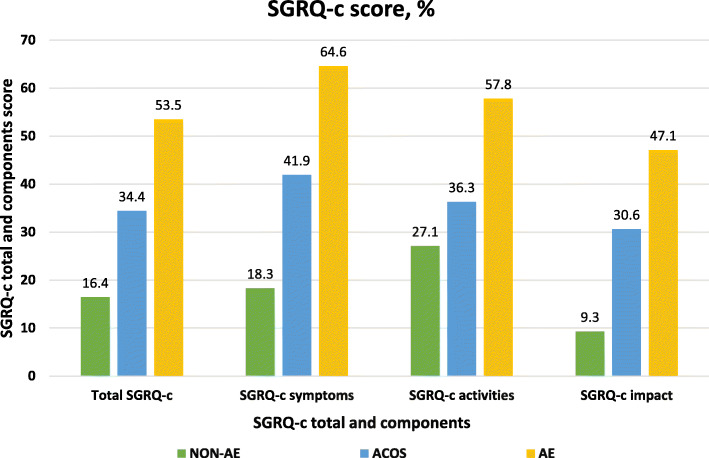
Score of SGRQ-c total and components according to COPD phenotypes. Abbreviation: SGRQ-c, St George’s Respiratory Questionnaire COPD; NON-AE, non-exacerbators; ACO, asthma-COPD overlap; AE, frequent exacerbators

Patients with AE had significantly higher score in each item of CAT and each component of SGRQ-c compared to those with NON-AE (all *p* <  0.001) (Fig. 2 and Fig. 3). Patients with AE also had significantly higher score in CAT 3 (*p* = 0.004), CAT 4 (*p* = 0.008), CAT 5 (*p* = 0.013), CAT 6 (*p* = 0.003) and CAT 8 (*p* = 0.016); as well as symptoms (*p* <  0.001), activities (*p* <  0.001), and impacts (*p* = 0.005) components of SGRQ-c, when compared to ACO patients. Compared to NON-AE patients, ACO patients had significantly higher score in each item of CAT (*p* = < 0.001–0.040) except CAT 2; as well as symptoms and impact components of SGRQ-c (*p* <  0.001).

The total CAT and SGRQ-c scores of the only nine AE NON-CB patients were significantly higher than that of NON-AE patients (12.6 ± 9.1 versus 5.5 ± 4.7, *p* = 0.018; 47.6 ± 18.5% versus 16.4 ± 14.8 <  0.001), but were not significantly different compared to those of AE-CB or ACO patients, respectively.

## Discussion

The most frequent COPD phenotype in this unselected population in the rural setting of Malaysia was NON-AE, followed by the AE-CB, ACO and AE NON-CB. Patients with AE were significantly older and smoked more cigarettes, while patients with ACO were predominantly female. Regardless of the COPD phenotypes, biomass fuel exposure was a common risk factor of COPD among them. Close to two-thirds of the patients were exposed to biomass fuel, mainly due to the seasonal open burning in agriculture activities.

The HRQoL of patients with AE and ACO was markedly impaired compared to normal individuals. Meanwhile, the HRQoL of patients with NON-AE was reduced when measured by SGRQ-c but not by CAT. The worst HRQoL was reported in patients with AE followed by those with ACO. The HRQoL of patients with AE was significantly worse than that of ACO and NON-AE while the HRQoL of ACO patients was significantly worse than the HRQoL of NON-AE patients. A similar pattern was also observed in each item of CAT and each component of SGRQ-c, except that the differences were not significant in cough, sputum, and sleep for AE versus ACO, as well as cough and daily activity limitation for ACO versus NON-AE. This lack of significance could be due to the smaller sample size of ACO, or the diurnal variation in symptomatology of bronchial asthma which is commonly associated with cough and sputum production.

The distribution of COPD phenotypes in the present study was almost similar to that of western populations, [24–27] except that AE NON-CB is less commonly reported than ACO [28]. So far, only two other studies have reported AE CB is the commonest COPD phenotypes followed by NON-AE, AE NON-CB and ACO. The first study was conducted in primary care centres of the Russia Federation, [29] while the second study involved selected COPD patients in the respiratory clinic of a tertiary hospital [30]. Our findings of patients with AE being older and smoked more cigarettes, [25, 27, 28, 31] as well as more female patients with the ACO phenotype are in agreement with other studies [24, 26–28]. The finding that the HRQoL of COPD patients was more impaired in the phenotype sequence of NON-AE, ACO and AE is consistent with the findings of previous studies [24, 26–28, 32]. Patients with AE are consistently highlighted as having the worst HRQoL, [24, 26–28, 31, 32] while those with NON-AE have the best HRQoL [25, 29]. Of patients with AE, Miravitlles et al., [28] Cosio et al., [31] Kania et al., [27] and Chai et al., [30] reported those with AE-CB have significantly worse HRQoL compared to other COPD phenotypes (all *p* <  0.001); while Corlatenau et al. reported the worst HRQoL in patients with AE NON-CB [32]. The CAT was uniformly used to asses HRQoL in these studies, with the latter two studies also using the SGRQ-c questionnaire. Only this study and that by Miravitlles et al., [28] show patients with ACO have significantly worse HRQoL than those with NON-AE.

Exacerbation is the prognostic hallmark of COPD. Frequent exacerbation is associated with poor HRQoL, [33] decline in lung function, [34] recurrence of exacerbations, [33] recurrent hospitalisations, [35] and increased mortality [36]. Seemungal et al. and Mackay et al., respectively reported COPD patients with ≥ three exacerbations (SGRQ-c, *p* <  0.001) and ≥ two exacerbations (CAT, *p* = 0.025) per year have significantly worse HRQoL [33, 37]. Cheng et al. also reported COPD frequent exacerbators have significantly worse HRQoL (mMRC, *p* <  0.001; CAT, *p* <  0.001) compared to non-frequent exacerbators [38]. Therefore, this explains the significantly worse HRQoL among our patients with AE. Despite similar exacerbation frequency, our patients with ACO had significantly worse HRQoL than those with NON-AE which highlights that COPD subtypes can also affect the patients’ HRQoL. Miravitlles et al. and Hardin et al., respectively reported COPD patients with BA have significantly worse HRQoL than those without [(mMRC, *p* = 0.008; SGRQ-c, *p* <  0.001), and (SGRQ-c, *p* = 0.008), respectively [39, 40]. Such a finding is not surprising in view of the presence of two different inflammatory processes in ACO.

The findings of our study support the recommendation of GesEPOC to phenotype every COPD patients based on their exacerbation frequency and COPD subtypes [15]. Besides, this study also highlights that exacerbation frequency supersedes COPD subtypes in determining the patients’ HRQoL. Therefore, clinicians should manage COPD patients with frequent exacerbations more aggressively, and consider prescribing pharmacotherapies such as long-acting muscarinic antagonist (LAMA), LAMA and long-acting ß_2_-agonist in combination, inhaled corticosteroids (ICS), roflumilast, macrolide, or N-acetylcysteine according to the COPD phenotype [1]. COPD treatment should also be personalised according to COPD subtypes, such as ICS for ACO, roflumilast for CB, and medical or surgical lung volume reduction for emphysema [1].

The present study is among the few in Asia that compares the HRQoL of COPD patients based on different clinical phenotypes. All the patients in this study were from the rural area. Their characteristics are very different from previous studies, such as having a high incidence of significant exposure to biomass fuel, required good physical fitness for agriculture activities, and had limited access to more expensive or newer COPD medications. Besides, we evaluated the HRQoL by using different HRQoL assessment tools and compared each of the subitem or component. By doing so we aimed to assess the patients’ HRQoL in more dimensions and to minimise biases.

There were several limitations in this study. Firstly, the number of AE NON-CB patients was disproportionally small and therefore we were unable to analyse it independently. We added AE NON-CB to AE-CB, and analysed in the line of AE for HRQoL analysis. Secondly, the direct comparison of CB versus emphysema subtypes was not possible because of the first limitation. Thirdly, the AE NON-CB phenotype was based on the finding of air-trapping on physical examination and on chest X-ray. Static lung volume measurement of functional residual capacity, residual volume and total lung capacity as well as non-contrast-enhanced thoracic computed tomography scan acquired at full inspiration and expiration that is able to differentiate emphysematous from non-emphysematous air-trapping were not performed [41]. Fourthly, spirometry used to identify COPD patients in this study utilised FVC_6_ instead of force vital capacity, potentially excluding a proportion of patients with mild COPD. Fifthly, body plethysmography and diffusion capacity for carbon monoxide (DLCO) were not performed. Studies have shown that body plethysmography and DLCO are more sensitive than spirometry in detecting early emphysema, evidenced by increase in residual volume and reduced DLCO [42]. Besides, COPD severity graded by using compression-free FEV_1_ measured by body plethysmograph is more accurate than FEV_1_ measured by spirometry [43]. Six, ACO in this study was defined based on a history of BA and very reversible airflow obstruction on spirometry testing. Blood eosinophil count was not routinely performed in the rural areas in Malaysia. The term ACO remains controversial without an agreed-upon definition [44]. Lastly, the exacerbation frequency was subjected to the recall error of the patients. We tried to minimize this error by confirmation from the patients’ medical records and with the patients’ family members.

## Conclusions

The present study concludes that HRQoL of patients with different COPD phenotypes is not the same. Patients with AE had the worst HRQoL, followed by those with ACO and NON-CB, respectively. The findings of this study support the recommendation of GesEPOC to phenotype and manage COPD patients based on their exacerbation frequency and COPD subtypes. COPD management should be personalised and more aggressive in frequent exacerbators to improve their poor HRQoL.

## Data Availability

The datasets used and/or analysed during the current study are available from the corresponding author on reasonable request.

## Abbreviations

COPD: Chronic obstructive pulmonary disease
HRQoL: Health-related quality of life
PB-FEV1: Post-bronchodilator forced expiratory volume in 1 s
PB-FVC_6_: Post-bronchodilator forced vital capacity in 6 s
mMRC: Modified Medical Research Council
CAT: COPD Assessment Test
SGRQ-c: St Georges Respiratory Questionnaire for COPD
PB-FEV_1_% predicted: PB-FEV_1_ in % of predicted
GesEPOC: Spanish COPD guideline
NON-AE: Non-exacerbator phenotype
AE: Exacerbator phenotype
AE CB: Exacerbator phenotype with chronic bronchitis
AE NON-CB: Exacerbator phenotype with emphysema
BA: Bronchial asthma
ACO: Asthma-COPD overlap phenotype
SD: Standard deviation
95% CI: 95% confidence interval
LAMA: Long-acting muscarinic antagonist
ICS: Inhaled corticosteroids
DLCO: Diffusion capacity for carbon monoxide
